# Plasmacytoid dendritic cells in systemic and cutaneous lupus erythematosus: an evolving understanding

**DOI:** 10.3389/fimmu.2026.1807771

**Published:** 2026-05-21

**Authors:** Victoria P. Werth, Lars Rönnblom, Joerg Wenzel, Margaret K. Moseley, Janine Gaiha-Rohrbach, Taylor L. Reynolds, Catherine Barbey, Jane L. Grogan, Marco Colonna

**Affiliations:** 1 Department of Dermatology, University of Pennsylvania, and Corporal Michael J. Crescenz VA Medical Center, Philadelphia, PA, United States; 2 Department of Medical Sciences, Rheumatology, Uppsala University, Uppsala, Sweden; 3 Department of Dermatology and Allergy, University Hospital Bonn, Bonn, Germany; 4 Biogen, Global Medical Affairs Immunology, Cambridge, MA, United States; 5 Biogen, Immunology Research Unit, Cambridge, MA, United States; 6 Clinical Development, Multiple Sclerosis and Immunology Department Unit, Biogen, Baar, Switzerland; 7 Department of Pathology and Immunology, Washington University in Saint Louis, Saint Louis, MO, United States

**Keywords:** cutaneous lupus erythematosus, plasmacytoid dendritic cells, systemic lupus erythematosus, targeted therapy, type I interferon

## Abstract

Lupus erythematosus is a chronic, heterogenous autoimmune disease driven by a complex interplay of genetic, environmental and hormonal factors, which can manifest as systemic lupus erythematosus (SLE), cutaneous lupus erythematosus (CLE), or both simultaneously. Plasmacytoid dendritic cells (pDCs) are thought to play a central role in disease pathogenesis following the dysregulated and sustained production of type I interferon (IFN-I), leading to widespread immune system activation and chronic inflammation, resulting in organ and tissue damage characteristic of lupus. While treatment of lupus has shown success with approaches that inhibit the IFN pathway/IFN-I production, targeting pDCs is of great interest due to their accumulation in lesional tissues and key role in IFN-I production. However, SLE and CLE pathogenesis is clouded by unknowns including limited understanding of the pathophysiological loss of IFN pathway negative feedback, the complexity of IFN production by non-pDC cells (including pDC plasticity and role of different subsets/states), the role of other cytokines and chemokines in disease pathogenesis, and differences in pDC activation and downstream effects in SLE and CLE. Conceivably, different IFN-I-producing cells may be important in different organs, patients, and disease stages. As understanding of the role of pDCs in IFN-I production evolves, there is great potential to develop more targeted and effective treatments that can enhance patient outcomes to ensure pathophysiological and chronic inflammation is negated while maintaining physiological immunity to infection.

## 1 Introduction

Systemic lupus erythematosus (SLE) and cutaneous lupus erythematosus (CLE) are multifactorial autoimmune diseases driven by a complex interplay of genetic, environmental, and hormonal factors resulting in activation of plasmacytoid dendritic cells (pDCs) and other cells of the immune system (1, 2). SLE is a potentially life-threatening condition characterized by multisystem involvement, affecting organs and tissues such as the skin, joints, kidneys, heart, lungs, and/or central nervous system (3). Autoantibody production, including antinuclear autoantibodies (ANAs), is a characteristic feature of SLE, which can be detectable years before clinical symptoms emerge (4). The clinical presentation of SLE is heterogeneous, commonly including symptoms such as arthritis and characteristic skin rashes (3, 5). Cutaneous involvement occurs in up to 85% of cases of SLE over the course of the disease (6). CLE primarily involves the skin and may occur as isolated CLE, or CLE with systemic manifestations (7). Approximately one-third of individuals with CLE either have a current diagnosis of SLE or will receive one at a future date (7). Clinical features of CLE include photosensitive rashes, scarring or dyspigmented lesions, alopecia, and mucosal ulcerations (8). Both SLE and CLE substantially impair health-related quality of life, stemming from pain, fatigue, and disfigurement due to recurrent skin lesions (7, 9–11). These factors contribute to psychological distress and negatively affect both physical and mental health domains (9–11).

Current standard of care therapies for SLE and CLE include antimalarials, glucocorticoids, and/or immunomodulators (**Table 1**) (12–19). Several biologic therapies for SLE have been developed to target key immune pathways implicated in SLE pathogenesis. Two approved biologics in SLE are belimumab, a monoclonal antibody that inhibits B-lymphocyte stimulator protein (also known as B-cell activating factor (20)), and anifrolumab, a monoclonal antibody that blocks type I interferon (IFN-I) receptor and inhibits IFN-I-mediated signaling (21). Although not approved for SLE treatment, rituximab, an anti-CD20 monoclonal antibody that depletes B cells, is used in selected SLE cases (particularly in refractory or organ-threatening disease) (12, 14, 22). Despite these therapeutic advances in SLE, there are currently no biologics or targeted drugs approved specifically for CLE (13, 23). While the approved treatments present favorable efficacy and safety profiles across a range of patients with SLE (24, 25), there is a significant proportion of patients who fail to respond or experience suboptimal treatment outcomes (5, 12). Consequently, it is necessary to develop therapies that efficiently target the appropriate components of the SLE and CLE pathogenesis pathways for effective disease control; by doing so, these drugs may help to improve treatment outcomes across patient populations (26). Expanding our knowledge on the pathogenesis of SLE and CLE would help to achieve this (2, 27).

**Table 1:** Current standard-of-care therapies for the treatment of SLE and CLE.

SLE	• Antimalarials (eg, hydroxychloroquine, chloroquine), corticosteroids (eg, prednisone), calcineurin inhibitors (eg, cyclosporine, tacrolimus, voclosporin), and immunomodulators (eg, methotrexate, mycophenolate, azathioprine, cyclophosphamide) (12) • Biologics: o Belimumab, a monoclonal antibody that inhibits BlyS (also known as BAFF) (approved in 2011 by the FDA and EC) (15, 16) o Anifrolumab, a monoclonal antibody that binds to IFNAR1 that mediates IFN-I signaling (approved in 2021 by the FDA and in 2022 by the EC) (17, 18) o Rituximab, an anti-CD20 monoclonal antibody that targets and depletes B cells (used off-label) (12, 14)
CLE	• Antimalarials, topical and oral corticosteroids, and immunomodulators, alongside recommendations for the use of calcineurin inhibitors, topical retinoids (eg, acitretin, isotretinoin), and antibiotics (eg, dapsone), optimized photoprotection, and avoidance of environmental triggers such as smoking (13, 19) • No biologics approved (13)

BAFF, B-cell activating factor; BLyS, B-lymphocyte stimulator; CD, cluster of differentiation; CLE, cutaneous lupus erythematosus; FDA, US Food and Drug Administration; EC, European Commission; IFN-I, type I interferon; IFNAR1, interferon α/β receptor 1; SLE, systemic lupus erythematosus.

Current understanding is that dysregulated and persistent IFN-I production can disrupt immune homeostasis, compromising self-tolerance and promoting autoantibody formation, which drives inflammation and contributes to the organ and tissue damage characteristic of SLE (28). Improved knowledge of the key drivers and sources of the pathophysiological IFN-I production will facilitate enhanced disease understanding. This review aims to critically examine the role of pDCs in the immunopathogenesis of SLE and CLE. We explore the phenotypical and functional heterogeneity of pDCs and emerging insights into additional cellular sources of IFN-I, and we outline key areas for future research that may inform the development of novel, targeted therapeutic strategies.

## 2 Overview of pDCs: characterization and function

pDCs have emerged as key players in the pathogenesis of SLE and CLE (29, 30), actively participating in early organ and tissue inflammation (31) and displaying distinct phenotypic and functional traits (**Table 2**) (28, 32–38). pDCs are a distinct lineage of immune cells generated in the bone marrow known for their capacity to produce large amounts of IFN-I in response to nucleic acid sensing, particularly during viral infections (39–41). IFN-I production in pDCs is mediated through Toll-like receptor (TLR) 7/9 expression (29). TLR7 and TLR9 are highly expressed endosomal nucleic acid-sensing receptors that recognize single-stranded RNA and unmethylated cytosine nucleotides with guanine motifs in DNA (29). Beyond endosomal sensing, human pDCs can be activated through cytosolic DNA detection, primarily via the cyclic guanosine monophosphate-adenosine monophosphate synthase (cGAS) and stimulator of IFN gene (STING) pathway (42). This mechanism operates alongside, and interacts with, endosomal TLR-mediated nucleic acid sensing (42).

**Table 2:** Summary of key characteristics of pDCs.

Characteristic	Description
Hematopoietic origin	pDCs develop from hematopoietic stem cells in the bone marrow (35)
Plasmacytoid morphology	pDCs typically have plasmacytoid morphology, although several subtypes with different morphologies have been identified (35, 37)
Migration to target tissues	pDCs migrate from the blood to peripheral and lesional tissues, where they contribute to immune responses (28, 35). pDC trafficking is context-dependent and mediated by surface markers, for example: CXCR4 (35); CCR7, CCR5, and L-selectin (35); CXCR3; and CXCL-chemokine interaction with CXCR3 (33)
Antigen presentation	pDCs can present viral antigens to T cells under certain conditions, which affects the adaptive immune response (34, 35)
IFN-I and IFN-III production	pDCs are major producers of IFN-I (including >12 IFNα subtypes and an IFNβ subtype), and IFN-III (whose receptor is restrictively expressed in cells of epithelial linage) in response to viral infections (32, 36, 38). Several pDC surface markers (eg, CD32a, BDCA2, or ILT7) and the endosomal receptors TLR7/9 mediate IFN-I production (35)

BDCA, blood dendritic cell antigen; CCR5/7, C-C motif chemokine receptor 5/7; CD, cluster of differentiation; CXCL, C-X-C motif chemokine ligand; CXCR, C-X-C motif chemokine receptor; IFN, interferon; IFN-I, type I interferon; IFN-III, type III interferon; ILT7, immunoglobulin-like transcript 7; pDC, plasmacytoid dendritic cell; TLR, Toll-like receptor.

After the initial IFN-I response, circulating pDCs migrate from the bloodstream to extra-lymphoid lesional tissues, such as inflamed skin or kidneys (28, 29, 43). Within these tissues, sustained pDC activation influences a broad spectrum of innate and adaptive immune cells, including monocytes, T cells, B cells, and other DCs, through direct interactions and signaling (28, 44). This activity establishes a positive feedback loop of IFN-I signaling (28). Under normal conditions, negative feedback mechanisms, both intracellular and at the cell surface, tightly regulate pDC IFN-I production to maintain immune balance (32). Indeed, IFN-I production by pDCs can be inhibited by surface marker blood dendritic cell antigen (BDCA) 2, a C-type lectin receptor that binds to glycoproteins such as serum immunoglobulins (29, 45). Inhibition of IFN-I production by BDCA2 is dependent on the presence of heparin and the activation status of pDCs. In the circulation, membrane-bound BDCA2 on pDCs binds to heparin, thereby suppressing TLR9-mediated IFN-I production. When pDCs migrate to the tissues and become activated, they have the capacity to release a soluble form of BDCA2 that can neutralize heparin, removing this inhibition and allowing IFN production to proceed; dysregulation of this mechanism may contribute to autoimmune disease (46). Additional surface markers on pDCs that regulate IFN-I production include BDCA4, immunoglobulin-like transcript 7 (ILT7; also known as leukocyte Ig-like receptor A4), sialic acid-binding immunoglobulin-like lectin H (Siglec-H), Fc gamma receptor IIa (FcγRIIa), CD123, and CD4 (29, 47–49).

Beyond IFN-I production, pDCs can produce type III interferons (IFN-III), a group of cytokines that are important for the skin and mucosal immune system to respond against viral infection (50). pDCs also mediate the production of other proinflammatory cytokines (eg, tumor necrosis factor [TNF] α, and interleukin [IL]-6/10/18 (32, 51)), and chemokines such as C-X-C motif chemokine ligand (CXCL) 8/9/10/11 (33, 41, 52, 53) and C-C-chemokine ligand (CCL) 3/4 (41). Additionally, once activated, pDCs express CD80 and CD86, and have costimulatory functions for T cells and B cells (41); human pDCs have been reported to cross-present exogenous soluble antigen to activate CD8+ T cells *in vitro* (34), although the impact of this function *in vivo* remains unclear (54). Another function of human pDCs is the production of granzyme B, a serine protease capable of inducing apoptosis via activation of caspases (55). pDC-derived granzyme B induces cytotoxic killing of keratinocytes by natural killer cells *in vitro*, with pDCs colocalizing with natural killer cells and cytotoxic T cells at areas of keratinocyte cell death *in vivo* (55). Furthermore, pDC depletion substantially reduced megakaryopoiesis and megakaryocyte numbers in mice (by 50% and 25%, respectively), suggesting that pDCs may be involved in the regulation of megakaryocytes (56).

## 3 pDCs are key players in the pathogenesis of SLE and CLE, and in other autoimmune diseases

### 3.1 Animal studies

Despite the differences in pDC surface markers between species (eg, Siglec-H in mice and CD123, BDCA2, and BDCA4 in humans), human and mammalian pDCs share high similarities, supporting the use of animal models to study lupus pathogenesis (57). In lupus-prone animal models, pDC depletion reduced TLR-mediated IFN-I production, prevented disease initiation, and/or slowed disease progression (58–61), and impairment of pDC function reduced disease severity (62). In a mouse model of TLR7- and TLR9-dependent skin inflammation, TLR7/9 inhibition led to reduced inflammatory cell infiltration and reduced pDC-mediated IFN-I production compared with normal mice (58). Additionally, pDC depletion with a specific antibody protected against glomerulonephritis in mice with lupus-like autoimmunity induced by TLR7-stimulation with imiquimod (60). pDC depletion at disease initiation in mice led to reduced levels of IFNα-induced gene transcripts, reduced levels of ANAs in serum, and fewer activated T cells and B cells (61). During the disease, the effect of pDC depletion was less pronounced in older mice compared with younger mice (aged 18 weeks and 4–12 weeks, respectively). In kidney glomeruli of the older mice, immunoglobulin G immune complex (IC) deposition was reduced despite minor effects on lymphocyte activation, indicating a role for pDCs in renal pathology (61).

In a mouse model of SLE engineered to express the diphtheria toxin receptor specifically in pDCs, transient pDC depletion was induced by administration of diphtheria toxin prior to disease onset, and its effects were assessed in mice at 19 weeks of age (59). pDC depletion resulted in impaired T-cell and B-cell expansion and activation, reduced antibodies against nuclear autoantigens, and improved kidney pathology, as well as reduced splenomegaly and lymphadenopathy. Although pDCs later recovered in older mice, pathological improvements were sustained. Furthermore, pDC function was impaired in two mouse models of SLE through the targeting of E protein transcription factor 4 (TCF4), leading to ameliorated SLE-like disease caused by TLR7 overexpression (62). Altogether, these results suggested that early transient pDC depletion ameliorated autoimmunity and that pDCs have a critical function in IFN-I-dependent initiation of SLE (59).

### 3.2 The role of pDCs in SLE and CLE

A broad range of human conditions including infectious diseases, malignancies, and autoimmune disorders are associated with a reduction in circulating pDCs and their concurrent accumulation in lymphoid and peripheral tissues (29, 63). Excessive pDC-dependent production of IFN-I has been described in several autoimmune diseases (29, 58), including Sjögren’s syndrome (64), dermatomyositis (65), systemic sclerosis (66, 67), vitiligo (68), psoriasis (69), several types of inflammatory arthritis (such as rheumatoid arthritis and spondyloarthropathy) (70, 71), SLE (50), and CLE (72). Human studies supporting the role of pDCs in SLE and CLE pathogenesis are described in **Table 3** (45, 48, 72–81). While useful information can be gleaned from these studies, many were exploratory and sample sizes were small. *In vitro* experiments showed pDCs in blood and skin lesions of patients with SLE and CLE (45, 48, 72–77). Clinical trials showed that reducing pDCs or inhibiting their IFN production was associated with decreased SLE and CLE disease activity (78–81).

**Table 3:** Evidence from human studies supporting the role of pDCs in the pathogenesis of SLE and CLE.

Study	Findings
de Vos et al. (2022) (72)	*In vitro* experiments showed pDC and B-cell infiltrates in skin lesions of patients with CLE
Vermi et al. (2009) (73)	*In vitro* experiments showed that there was pDC infiltration in the skin lesions of patients with SLE and CLE
Murayama et al. (2017) (74)	*In vitro* experiments showed that exposure to IFNα enhanced IFNα production upon TLR7 stimulation in pDCs from patients with SLE. The IFNα-producing capacity of pDCs was associated with disease activity and endogenous serum IFNα levels
Pellerin et al. (2015) (75)	*In vitro* experiments showed that litifilimab inhibited TLR-induced IFN-I production in pDCs from the peripheral blood of both patients with SLE and healthy controls
Walsh et al. (2015) (76)	*In vitro* experiments showed that pDCs were present in inflammatory skin lesions of patients with hypertrophic DLE
Blomberg et al. (2003) (48)	*In vitro* experiments reported a reduced number of BDCA2–expressing pDCs in the blood of patients with SLE, and inhibition of their IFNα production by anti-BDCA2 and anti-BDCA4 monoclonal antibodies
Dzionek et al. (2001) (45)	*In vitro* experiments showed that ligation of BDCA2 with a monoclonal antibody potently suppressed induction of IFNα/β production in pDCs
Blanco et al. (2001) (77)	*In vitro* experiments highlighted that, although there were reduced numbers of CD11c^−^ CD123^+^ pDCs in the blood of patients with SLE, cells from this pDC subset produced normal levels of IFNα in response to viral triggering
Furie et al. (2022) (78)	A phase 2 trial showed that litifilimab led to a greater reduction in the number of swollen and tender joints, and active skin disease (measured by SRI-4), and improvements in overall disease activity (measured by SLEDAI-2K) versus placebo in patients with SLE
Werth et al. (2022) (79)	A phase 2 trial showed that litifilimab was superior to placebo in reducing skin disease activity in patients with CLE
Karnell et al. (2021) (80)	A phase 1 trial showed that a monoclonal antibody targeting ILT7 reduced both circulating and tissue-resident pDCs in patients with CLE, and that reductions in pDCs in the skin correlated with a decrease in local IFN-I activity and improvements in clinical disease activity
Furie et al. (2019) (81)	A phase 1 trial showed that litifilimab reduced the expression of IFN response genes in blood, normalized MxA expression, reduced immune infiltrates in skin lesions, and decreased CLASI-A score in patients with SLE

BDCA, blood dendritic cell antigen; CD, cluster of differentiation; CLASI-A, Cutaneous Lupus Erythematosus Disease Area and Severity Index Activity; CLE, cutaneous lupus erythematosus; DLE, discoid lupus erythematosus; IFN, interferon; IFN-I, type I interferon; ILT7, immunoglobulin-like transcript 7; MxA, myxovirus resistance protein A; pDC, plasmacytoid dendritic cell; SLE, systemic lupus erythematosus; SLEDAI-2K, Systemic Lupus Erythematosus Disease Activity Index – 2000; SRI-4, Systemic Lupus Erythematosus Responder Index – 4 TLR, Toll-like receptor.

**Figure 1:**
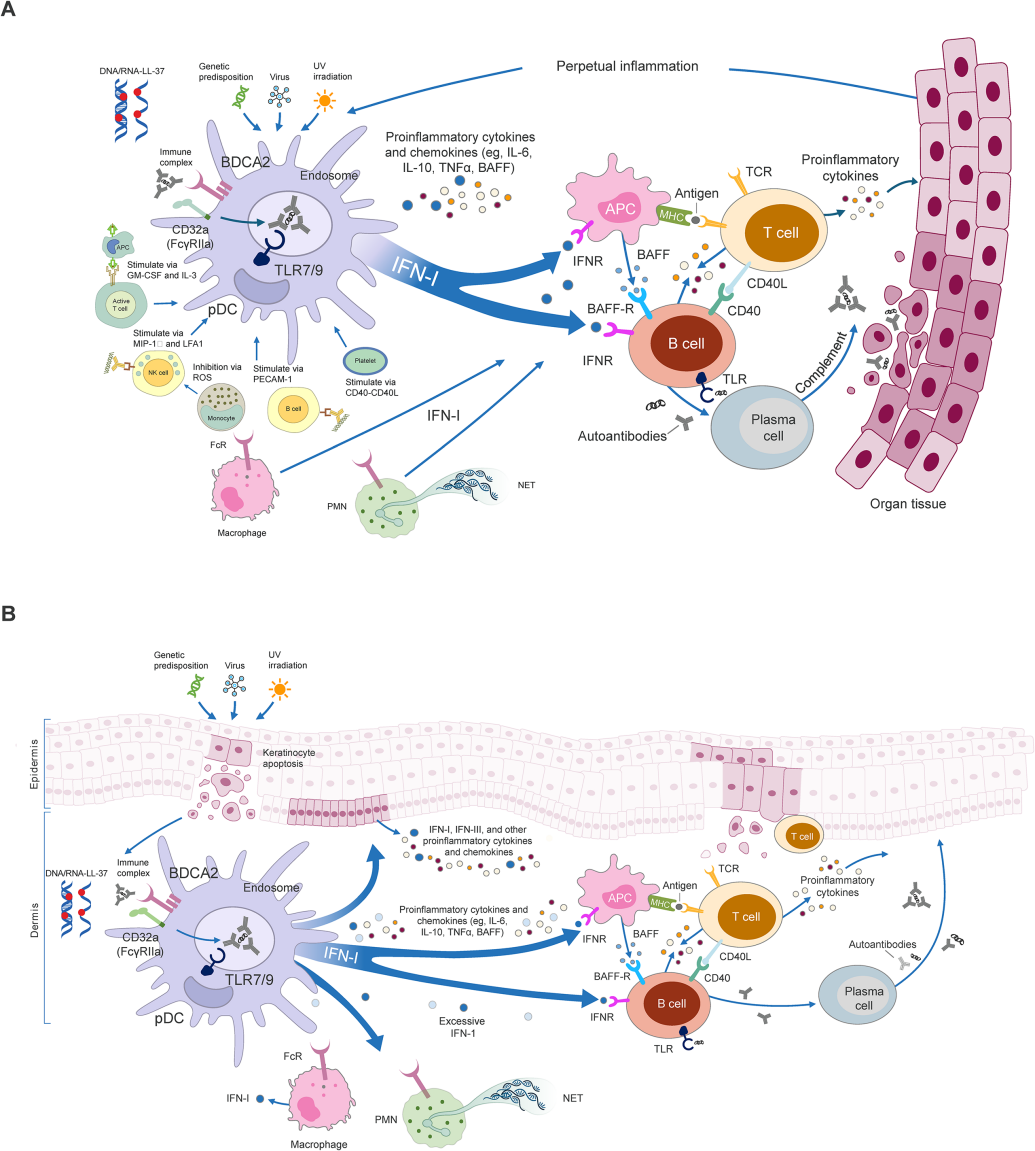
Immune pathways involving pDCs in **(A)** SLE and **(B)** CLE. **(A)** In SLE, environmental triggers (eg, UV light or viruses) generate nucleic acids that form ICs, including AMP-nucleic acid complexes such as DNA/RNA-LL37 (1, 28, 82). ICs bind to CD32a on the surface of pDCs, are internalized, and activate TLR7/9 pathways that upregulate the production of IFN-I and other proinflammatory cytokines and chemokines. Conceivable promoters of IFN production in pDCs include IC-activated NK cells and B cells, which stimulate pDCs via MIP-1β and PECAM-1, respectively. Additionally, platelets (via CD40-CD40L) and activated T cells may also stimulate pDCs via GM-CSF and IL-3 (28). Immune cells, including macrophages and neutrophils, can also be sources of IFN-I production in SLE (28). IFN-I activates APCs, which promote cytokine production and tissue damage through the activation of autoreactive T cells (14, 29). IFN-I and T-cell interactions drive B-cell activation and differentiation into plasma cells (14, 36). This leads to the production of high levels of autoantibodies, resulting in complement activation and additional formation of ICs, fueling perpetual inflammation and organ and tissue damage (14). **(B)** In CLE, UV irradiation can cause cytokine release and keratinocyte apoptosis, generating cell degradation products, such as nucleic acids, that form ICs, including AMP-nucleic acid complexes such as DNA/RNA-LL37 (31, 88). As in SLE, these ICs activate the TLR7/9 pathway leading to an upregulated production of IFN-I and other proinflammatory cytokines and chemokines. IFN-I signaling in damaged keratinocytes prompts further recruitment of pDCs, T cells, and macrophages into lesional skin. T-cell and B-cell interactions, as well as IFN-I production from other immune cells including macrophages and neutrophils, further contribute to inflammation and skin lesions, forming a positive feedback loop of cytokine release, keratinocyte apoptosis, and pDC activation (30, 31, 43). AMP, antimicrobial peptide, APC, antigen-presenting cell; BAFF, B-cell activating factor; BAFF-R, B-cell activating factor receptor; BDCA2, blood dendritic cell antigen 2; CD, cluster of differentiation; CD40L, cluster of differentiation 40 ligand; CLE, cutaneous lupus erythematosus; FcR, Fc receptor; FcγRIIa, Fc gamma receptor IIa; GM-CSF, granulocyte-macrophage colony-stimulating factor; IC, immune complex; IFN, interferon; IFN-I, type I interferon; IFN-III, type III interferon; IFNR, interferon receptor; IL, interleukin; LFA1, lymphocyte function-associated antigen 1; MHC, major histocompatibility complex; MIP-1β, macrophage inflammatory protein-1 beta; NET, neutrophil extracellular trap; NK, natural killer; pDC, plasmacytoid dendritic cell; PECAM-1, platelet endothelial cell adhesion molecule-1; PMN, polymorphonuclear neutrophil; ROS, reactive oxygen species; SLE, systemic lupus erythematosus; TCR, T-cell receptor; TLR, Toll-like receptor; TNFα, tumor necrosis factor α; UV, ultraviolet.

In SLE, ICs containing nucleic acids and small nuclear ribonucleoproteins are recognized and internalized by pDCs via CD32a (FcγRIIa) (47, 75). Once internalized, the nucleic acid components engage TLR7/9, initiating IFN-I production (**Figure 1A**) (1, 14, 28, 29, 36, 47, 75, 82). Factors such as microbial agents, neutrophil extracellular traps (NETs), and mitochondria can also participate in pDC activation (29). A study showed that, in the cytosolic compartment, expression of the cGAS–STING pathway is increased in a proportion of patients with SLE and promotes IFN-I production, although further research is needed to clarify the differential roles of STING in SLE pathogenesis (83). pDC activation via the cGAS–STING pathway may also play an important role in CLE because cGAS-dependent photosensitivity and the subsequent production of IFN-I have been observed after exposure to ultraviolet light in patients with lupus (84).

The persistent activation of IFN-I signaling pathways occurs because pDCs escape negative feedback mechanisms that regulate their IFN-I production in a way that is not fully understood (85). pDC activation may also affect a variety of cell types via downstream effects of the IFN-I and IFN-III pathways, as well as through T–B-cell interactions that lead to autoantibody production (28, 35, 36). For example, elevated levels of IFN-I (specifically IFNα) can occur in multiple tissues following pDC accumulation. Recent evidence indicates that pDC generation and activation are regulated by redox balance, whereby neutrophil cytosolic factor 1 (NCF1)-mediated reactive oxygen species (ROS) restrains pDC activation (86). Reduced ROS, due to NCF1 deficiency or polymorphisms, promotes pDC accumulation in multiple organs and excessive IFNα production, aggravating lupus (86).

In SLE and CLE, pDCs have been found to accumulate in lesional skin and have been associated with high IFN-I expression at these sites (72, 73). Although pDCs are central to the pathogenesis of both SLE and CLE (31), the signaling pathways and cellular interactions involved differ. In CLE, tissue inflammation can occur independently of B cells (22, 72), and pDC activation is shaped by local mediators such as Langerhans cells (specialized DCs in the epidermis) and keratinocytes (the resident epithelial cells) (**Figure 1B**) (30, 31, 43, 87). These keratinocytes, along with other immune cells, engage in dynamic crosstalk with pDCs (28, 31, 88), contributing to the inflammatory milieu. pDCs and autoreactive T and B cells all play an important role in the pathogenesis of SLE and CLE (89, 90). The characteristic loss of adaptive immune tolerance results in high levels of autoreactive B cells, leading to increased signaling from TLR ligands and T-cell-derived cytokines (89). High levels of autoantibody production cause inflammation and tissue and cell damage (89).

## 4 pDCs exhibit heterogeneous phenotypes and functions

pDCs present as several phenotypically and functionally different subsets (35), which have been described in humans (**Table 4**) (35, 37, 91–95). Two phenotypically and functionally distinct IFNα-producing pDC subsets were initially described in blood and in secondary lymphoid organs based on CD2 expression: CD2^high^ pDCs, characterized by prevalent expression of myeloid-related genes and strong capacity to initiate T-cell immune responses; and CD2^low^ pDCs, originating from myeloid hematopoietic progenitor cells, and with less capacity to induce naïve allogeneic T-cell proliferation (ie, expansion of T cells that did not previously encounter a foreign antigen) compared with CD2^high^ pDCs (91). A pDC subset overlapping with CD2^high^ pDCs was subsequently shown to mediate IFN-I-independent maintenance of immune homeostasis (92). In blood, bone marrow, and tonsil samples of healthy volunteers, this pDC subset expressed CD5 and CD81 and did not produce IFN-I upon stimulation, but did release large quantities of other proinflammatory cytokines that potently induced plasma cell formation and antibody secretion (92). It was recently shown that CD2^high^ pDCs in fact correspond to a distinct DC population that expresses Axl receptor tyrosine kinase, sialic acid-binding immunoglobulin-like lectin 6 (Siglec-6), and a continuum of pDC and conventional DC2 (cDC2) markers (35, 93). These “AS DCs” share properties with pDCs such as BDCA2 expression and are capable of initiating T-cell responses (35, 93).

**Table 4:** Summary of select pDC subsets (observed to express BDCA2) that have been described in previous research.

Surface marker	Description
CD2, CD5, and CD81	• CD2^high^ pDCs: myeloid origin, potent initiators of T-cell immune responses (91) • CD2^low^ pDCs: lymphoid origin, non-potent initiators of T-cell immune responses (91) • CD2^high^ pDCs: two subsets based on expression of CD5 and CD81. CD5^−^CD81^−^, produce large amounts of IFN-I; CD5^+^CD81^+^ produce no IFN-I, but compared with CD5^−^CD81^−^ they secrete increased amounts of proinflammatory cytokines and chemokines (92)
CD123, CD45RA	• CD123^high^ pre-DC subset (an early uncommitted subset) (94) • CD45RA^+^ CD123^low^ DC (two lineage-committed DC subsets exhibiting functional differences) (94) • CD1a^+^ CD123^int^ DC (with antigen-presentation capabilities) (95)
PDL1, CD80	• P1 PDL1^+^ CD80^−^: plasmacytoid morphology, IFN-I production (37) • P2 PDL1^+^ CD80^+^: intermediate morphology (plasmacytoid dendritic), limited IFN-I production (37) • P3 PDL1^−^ CD80^+^: dendritic morphology, no IFN-I production (37)
AXL	• AXL^+^ DCs: shared properties with pDCs such as BDCA2 expression, initiators of T-cell responses (35, 93)

AXL, Axl receptor tyrosine kinase; BDCA2, blood dendritic cell antigen 2; CD, cluster of differentiation; DC, dendritic cell; IFN-I, type I interferon; pDC, plasmacytoid dendritic cell; PDL, programmed death ligand.

Human pDCs were also reported to diversify into three stable states in response to viral infection with diverse phenotypes based on differential expression of programmed death ligand (PDL) 1 and CD80 (37). One of these pDC states, defined as P1 (PDL1^+^ CD80^−^), displayed plasmacytoid morphology and was specialized for IFN-I production; 80% of IFNα-producing pDCs had this phenotype. The other two pDC states represented a smaller proportion of IFNα-producing cells: P2 represented 15% of IFNα-producing cells (PDL1^+^CD80^+^), which had a morphology intermediate between plasmacytoid and dendritic, displayed both innate and adaptive functions; and P3 represented 1% of IFNα-producing cells (PDL1^−^CD80^+^), which adopted dendritic morphology and adaptive immune functions. This study also showed the presence of a P1-like population in blood from patients with lupus, and a large P1 cell population, but few P2 cells and no P3 cells, in the skin of patients with active psoriasis (37). Consistent with these results, in a mouse model it was observed that pDCs can produce IFN-I and activate T cells, but sequentially and in different micro-anatomical locations (96). A recent study has shown that, when activated, fully differentiated pDCs can undergo fate switching, promoted by TNF and blocked by IFN-I, resulting in a cDC2-like identity and function (97). Similarly, cDC2 differentiation potential has been observed in transitional DCs, which are a distinct pDC-related subset with proinflammatory potential during viral infection (98).

Of note, human DC precursors in the bone marrow were shown to present distinct subpopulations expressing BDCA2, which do not correspond to bona fide pDCs: an uncommitted lineage subset (CD123^high^) and two lineage-committed subsets expressing an isoform of CD45 (CD45RA^+^ CD123^low^) (94). Another study identified a DC subset (CD123^int^ BDCA2^+^ CD1a^+^) that mimicked pDCs and infiltrated human skin wounds during acute sterile inflammation, acquiring features compatible with lymph node homing and antigen presentation (95). Thus, not all BDCA2^+^ cells represent bona fide pDCs; instead, multiple distinct DC subsets can express BDCA2 and adopt pDC-like features depending on their developmental stage or inflammatory context, highlighting the functional and phenotypical diversity in the human DC lineage. Altogether, these findings demonstrate the complexity and plasticity of pDC fate and function (38, 96, 97), opening new research paths to better understand the role of pDCs in autoimmune diseases such as SLE and CLE.

## 5 Characterization of pDC infiltrates may help to diagnose lupus skin lesions

**Figure 2:**
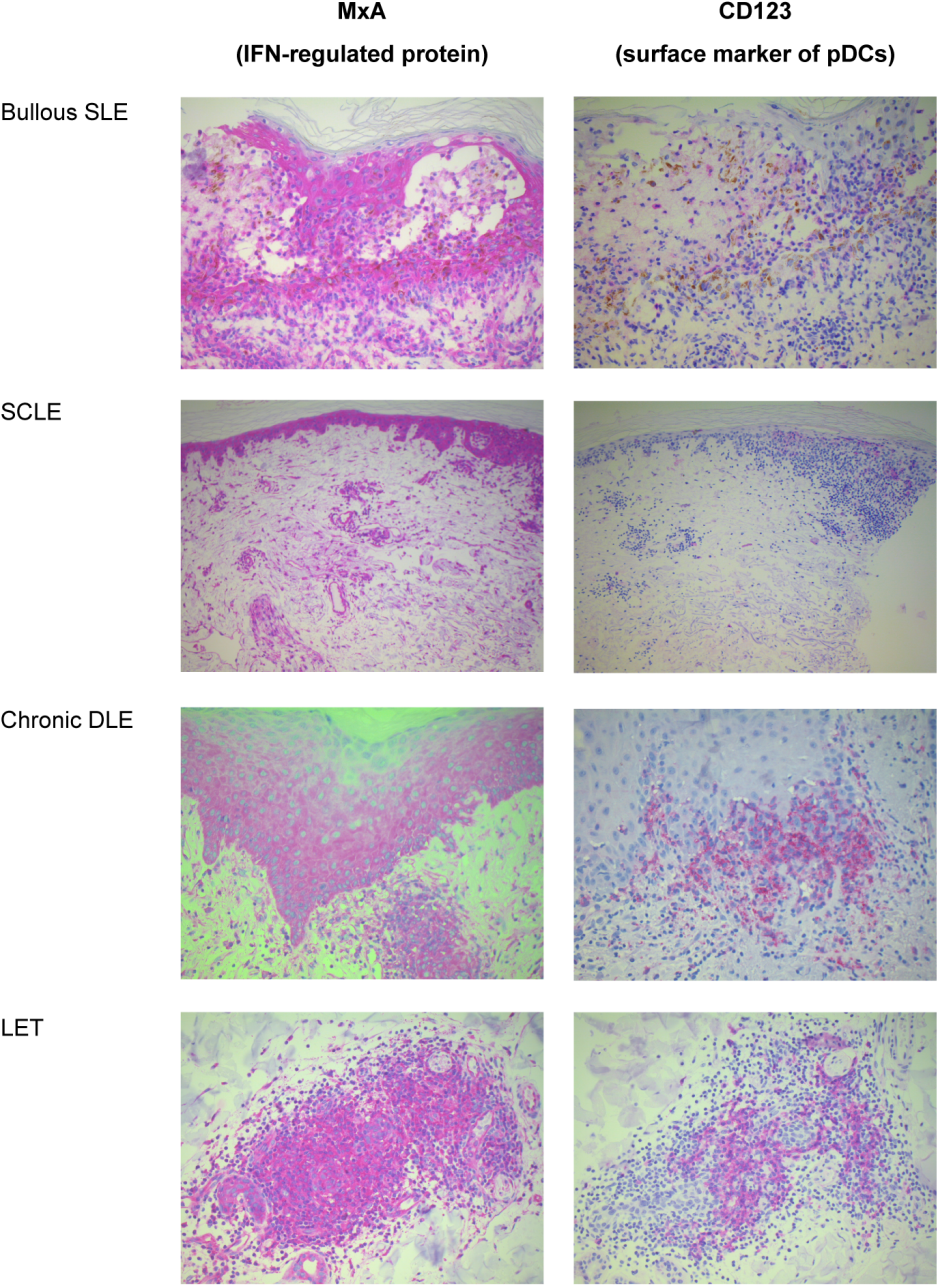
Images of immunohistochemical staining (red) of skin biopsies of patients with SLE or CLE showing an IFN-modulated protein accompanied by the presence of pDCs.^a. a^Images provided by Dr Joerg Wenzel; the observations were made through the detection of MxA (an IFN-I-/IFN-III-regulated protein) and CD123, a surface marker for pDCs (72, 101). Immunohistochemistry was conducted using the ZytoChem Plus AP Polymer System (mouse/rabbit) with Zytomed AP-Red Kit following the manufacturer’s instructions (Zytomed Systems GmbH, Berlin, Germany). The following monoclonal antibodies were used: MxA (M143 University Medical Centre Freiburg, Germany, 1 mg/mL); and CD123 (BD biosciences, New Jersey, USA, 1:200 dilution, pH 9.0). All antibodies were diluted with Zytomed Antibody Diluent (ZUC025-100; Zytomed Systems GmbH, Berlin, Germany). Staining was performed using the DAKO-OMNIS (Agilent) machine. CD, cluster of differentiation; CLE, cutaneous lupus erythematosus; DLE, discoid lupus erythematosus; IFN, interferon; IFN-I, type I interferon; IFN-III, type III interferon; LET, lupus erythematosus tumidus; MxA, myxovirus resistance protein A1; pDC, plasmacytoid dendritic cell; SCLE, subacute cutaneous lupus erythematosus; SLE, systemic lupus erythematosus.

In the skin of patients with SLE or CLE, pDCs accumulate either in the dermis or at the dermal-epidermal junction (DEJ) (99), with high pDC infiltration in skin lesions accompanied by strong IFN-I expression (58, 72, 73, 100). Author-generated images of pDC infiltration in skin lesions of patients with bullous SLE (an uncommon cutaneous manifestation of SLE (7)), or with diagnosed CLE subtypes (subacute cutaneous lupus erythematosus [SCLE], discoid lupus erythematosus [DLE], or lupus tumidus) are provided in **Figure 2** (72, 101).

Biopsies of inflammatory skin lesions from patients with CLE exhibit large clusters of at least 40 CD123^+^ cells per field (representing 10% of the overall infiltrate) in 71% of patients with SCLE, and in 59% of patients with DLE (100). Additionally, the distribution of pDCs in patients with SCLE and DLE was superficial; for almost all SCLE cases, the distribution was superficial at the DEJ and within the epidermis, as in patients with other autoimmune diseases such as dermatomyositis (100). pDC clusters were more frequent and larger in CLE compared with other inflammatory diseases (eg, rosacea) (100), and the skin of patients with other diseases did not present pDC clusters (eg, subcutaneous panniculitis-like T-cell lymphoma) (102). A dense band of CD123+ cells at the DEJ was observed in hypertrophic lupus erythematosus, a variant of DLE, which differed from findings in the skin of patients with squamous cell carcinoma or actinic keratoses, in whom CD123+ cells appeared as single cells or in scattered clusters (103). Three criteria identified as having diagnostic value in patients with hypertrophic lupus erythematosus were pDC representation of at least 10% of the inflammatory infiltrate, over ten cells arranged in clusters, and presence at the DEJ (76). It should be noted that CD123 is not a unique marker of pDCs, being expressed by other myeloid cells such as basophils and monocytes (104), and therefore additional analyses should be conducted to confirm the nature of cells expressing CD123. These results warrant further research on the characterization of pDC infiltrates in inflammatory skin lesions as a diagnostic tool for lupus skin lesions.

## 6 pDCs are potential therapeutic targets in the IFN pathway

The importance of the IFN pathway in the pathogenesis of SLE and CLE is reinforced not only by preclinical studies, but also by the clinical benefit observed with drugs that interfere with one of the components of the IFN pathway, or that interfere with pDCs directly (**Table 5**) (7, 17, 18, 75, 80, 81, 85, 105–113). For example, the antimalarials hydroxychloroquine, chloroquine, and quinacrine, which are used in SLE and CLE treatment, were shown to reduce IFNα production via an inhibitory effect on TLR activation (105, 106). Studies on deucravacitinib (tyrosine kinase 2 [TYK2] inhibitor in the IFN signaling pathway) have provided additional supportive data; in a phase 2 trial in patients with active SLE, deucravacitinib reduced IFN-I-regulated gene expression and increased response rates for several outcome measures, including the Cutaneous Lupus Erythematosus Disease Area and Severity Index (CLASI), an assessment of skin disease activity in CLE (107). Additionally, TLR recognition of self-DNA/RNA is an important inflammatory amplifier in patients with SLE (114), with continued TLR7 and TLR9 stimulation by circulating ANAs inducing IFN-I production. A study of stimulated human peripheral blood mononuclear cells (PBMCs) from healthy donors treated with enpatoran (a potent and selective dual TLR7/8 inhibitor), dexamethasone, or both, showed that combination treatment inhibited IL-6 release and reduced the expression of nuclear factor kappa B (NF-κB) and IFN-regulated genes (108). In the context of biologics, anifrolumab downregulated IFN-I-induced genes (115) and improved clinical outcomes in patients with SLE (including patients with cutaneous manifestations) in phase 2 and 3 trials (116, 117). In a phase 3 trial (117), anifrolumab showed a reduction in moderate-to-severe disease activity in approximately 48% of patients who received the drug, as measured by the achievement of a response in the British Isles Lupus Assessment Group–based Composite Lupus Assessment (BICLA).

**Table 5:** Standard of care and investigational drugs that reduce IFN-I production by interfering with components of the IFN pathway.

Drug	Mode of action	Usage^a^
Standard of care and investigational drugs that interfere with the IFN pathway and IFN-I production		
Hydroxychloroquine (40, 106)	Antimalarial that inhibits pDC-mediated IFN-I production by preventing TLR activation	SLE, CLE
Quinacrine (7, 105)	Antimalarial that colocalizes with ICs in the endosomes, where it blocks TLR activation	SLE, CLE
Anifrolumab (17, 18)	Monoclonal antibody that binds to IFNAR1 that mediates IFN-I signaling	SLE
Deucravacitinib (107, 111, 112)	Small molecule that targets the regulatory domain of TYK2 to selectively inhibit IL-12, IL-23, and IFN signaling	SLE, CLE
Enpatoran (108, 113)	Small molecule that acts as a potent and selective dual TLR7/8 inhibitor of IFNα production	SLE, CLE
Investigational drugs that deplete pDCs or inhibit their production of IFN-I		
Daxdilimab (80, 109)	Monoclonal antibody that depletes pDCs by binding to ILT7	SLE, CLE
Talacotuzumab (110)	Monoclonal antibody that depletes pDCs by binding to CD123	SLE
Litifilimab (75, 81)	Monoclonal antibody that inhibits TLR-induced IFN-I production by binding to BDCA2	SLE, CLE

BDCA, blood dendritic cell antigen; CD, cluster of differentiation; CLE, cutaneous lupus erythematosus; IC, immune complex; IFN, interferon; IFN-I, type I interferon; IFNAR1, interferon α/β receptor 1; IL, interleukin; ILT7, immunoglobulin-like transcript 7; pDC, plasmacytoid dendritic cell; SLE, systemic lupus erythematosus; TLR, Toll-like receptor; TYK2, tyrosine kinase 2 inhibitor.

^a^ Based on clinical trial data and/or regulatory approvals.

As a source of IFN-I, targeting pDCs has been shown to be promising. In phase 1 trials, daxdilimab (anti-ILT7 monoclonal antibody [mAb]) reduced both blood- and tissue-resident pDCs in patients with CLE, with an approximately 98% reduction in the skin after 85 days of treatment, correlating with a decrease in local IFN-I activity and improvement in clinical disease activity (80). In a phase 2 trial in patients with SLE, daxdilimab did not meet its primary endpoint in reducing disease activity, as measured by BICLA response while lowering daily glucocorticoid dose, but it was associated with stable, low disease activity (measured by the achievement of improved Lupus Low Disease Activity State [LLDAS]) (109). Additionally, talacotuzumab (anti-CD123 mAb) depleted pDCs *in vitro* and ablated SLE-IC-induced IFN responses in a small study of 33 patients (110).

Previous studies showed that complete pDC depletion affects antiviral immunity, and that pDCs may reach an “exhausted” state during chronic viral infection that impairs the host’s ability to combat secondary infections (75, 118); therefore, therapeutic strategies aiming to achieve an optimal modulation of pDC function, instead of causing pDC depletion, may help to reduce infection susceptibility. The possibility for pDC inhibition with an anti–BDCA2 mAb was first demonstrated *in vitro* (45), and was later validated by an *in vitro* study of PBMCs from patients with SLE (48).

More recently, the anti–BDCA2 mAb litifilimab was shown to inhibit TLR-induced IFN-I production in pDCs from peripheral blood of both patients with SLE and healthy controls (75). In addition, ex-vivo analyses of litifilimab-treated pDCs from healthy controls showed inhibition of IFN-I production as well as of other pDC-derived proinflammatory mediators, including IL-6, TNFα, CCL3, CCL4, CCL5, and IFNλ1 (81). Further research demonstrated that targeting BDCA2 leads to functional inactivation of pDCs in a xenotransplant mouse model, with suppression of the entire TLR9-induced transcriptome, including IFN-I activation and a multitude of genes that could contribute to immune-driven skin inflammation and fibrosis (119). In a phase 2 trial, compared with placebo, litifilimab demonstrated a greater reduction in the number of swollen and tender joints in patients with SLE and active skin disease and joint involvement (78). Litifilimab also reduced skin disease activity in participants with active CLE (including SCLE and/or chronic CLE, including DLE), with or without SLE, compared with placebo (79). The efficacy and safety of litifilimab are currently under evaluation in a phase 2/3 trial in CLE and phase 3 trials in SLE (120–124).

## 7 Beyond pDCs: other cells as key contributors to IFN-I production

In recent years, research has challenged the assumption that pDCs are the main drivers of IFN-I production in the pathogenesis of SLE and CLE (55, 125, 126). In SLE, it was observed that pDCs present a transcriptional signature indicative of cellular stress and senescence accompanied by increased telomere erosion (125). Senescence in pDCs is associated with loss of immunogenic functions (125), including reducing their ability to activate the adaptive immune system (126). In SLE, a large proportion of pDCs, higher than in healthy individuals, fail to produce IFN after stimulation, whereas the remainder of the pDCs appear capable of synthesizing normal amounts (127). Additionally, pDCs from patients with SLE were observed to reduce their capacity to induce TLR-mediated production of IFN-III, TNF, and IL-10, and did not induce T-cell proliferation and activation, independent of disease activity and IFN signature in the blood (125). However, these findings may be confounded by the high proportion of patients that were taking hydroxychloroquine (83%) (125), which may impair the TLR-mediated responses in pDCs (128). In treatment-naïve patients with CLE, both skin-localized and circulating pDCs were found to express IFNα upon TLR7 stimulation, but this was reduced compared to healthy controls (55).

Besides pDCs, other cells may be involved in IFN-I production. Several types of leukocytes (CD68+/M1/M2 macrophages, and CD16+ cells) were shown to express, after stimulation, more IFNα in the skin of patients with CLE compared with healthy controls (55). Nonhematopoietic cells such as fibroblasts (stromal cells) were observed to present a high IFNα signature in nonlesional skin of patients with CLE (126), and keratinocytes have been found to mediate IFN-I production (125). A study reported a strong IFN response signal (measured via the expression of myxovirus resistance protein A1 [MxA], an IFN-regulated protein) in skin cells with low or absent pDC infiltration in patients with SCLE and chronic CLE (including DLE) (72). Similar to pDCs, keratinocytes are also capable of IFN-III production (28). In the epidermis of CLE lesions, IFN-III induces the production of other proinflammatory cytokines and chemokines such as CXCL9/10/11 (99). Deficiency of the IFN-III receptor led to reduced skin inflammation in a lupus mouse model (129).

Markedly, an IFN-I-rich signature was found in both lesional and nonlesional skin of patients with CLE (126). This IFN-I-rich signature influenced transcription and intercellular communication of various skin cell types and CD16+ DCs (a myeloid cell subset implicated in the pathogenesis of lupus), shifting them to a proinflammatory phenotype. CD16+ DCs in the skin underwent robust IFN education (ie, enhancement of responsiveness to stimuli), with proficient intercellular communication even in nonlesional skin in which they were highly abundant. Most IFN education occurred at the DEJ, where keratinocytes and immune cells (eg, CD16+ DC and pDCs) primarily localized. On the other hand, myeloid cell subsets were observed to shift depending on skin lesional state, with pDCs dominating in nonlesional skin (126). However, despite their predominance, pDCs appeared largely inactive compared with CD16+ DCs, and this evidence supported that CD16+ DCs have a role in the pathogenesis of CLE, and a potential role of IFN-I in disease initiation. CD16+ DCs were also shown to present higher interaction in ligand-receptor analyses in contrast to pDCs, which rarely participated in ligand-receptor pairs and remained inert in nonlesional skin of patients with CLE (126).

Thus, several different cell types, besides pDCs, can contribute to the ongoing interferon production in SLE, and different interferon-producing cells may be important in different organs, different patients, or at various stages of the disease.

## 8 Limitations of previous research and future research needs

There are several limitations and unanswered questions in the research of the pathogenesis of SLE and CLE. An inherent limitation of research using animal models is that animal pDCs present characteristics and functions that may not be fully transferrable to human pDCs. For instance, TLR7/9 are primarily expressed in pDCs and B cells in humans, while in mice TLR7/9 are also expressed in most myeloid cells (130).

SLE has high clinical heterogeneity across patients (7, 131), with most patients exhibiting cutaneous involvement during the disease course (6). CLE itself may occur as an isolated condition or alongside systemic manifestations (7). This diversity in SLE and CLE may obscure the role of pDCs in the pathogenesis of each of these indications. Additionally, pDC responsiveness may be underreported in studies including patients who have received treatment at the time of inclusion. For instance, the study by Billi et al, which evaluated skin transcriptional changes leading to CLE, included only one patient with CLE without concomitant SLE, and treatment most commonly included hydroxychloroquine (126). The heterogeneity in pDC subsets and their characteristics based on their location in blood versus target tissues may also challenge the interpretation of the role of pDCs in the pathogenesis of SLE and CLE. Recent studies focusing on therapies that target pDCs in CLE specifically may help to clarify further the role of pDCs in the pathogenesis of CLE and the differences from their role in SLE (79, 132).

Experimental techniques also present some limitations. The type of gene expression measured by investigators may contribute to inconsistencies in the results obtained across studies because there is overlap between genes induced by different IFN types (133). Notably, our understanding of pDC function mainly relies on transcriptomic data (101). Single-cell RNA sequencing (scRNA-seq) combined with immunohistochemistry allows the analysis of tissue samples at the cellular level and measurement of median signal expression at the protein level (55); however, not measuring messenger RNA expression across entire biopsies (55) may miss patterns of gene expression involved in pathogenic characteristics (eg, inflammation). In addition, scRNA-seq provides fewer reads per gene per cell than bulk RNA sequencing methods (134), which may preclude direct examination of transcript levels for many cytokines, particularly IFNs (126). Moreover, the isolation of skin cells such as keratinocytes or fibroblasts for scRNA-seq, which involves processing steps that can introduce bias in cell recovery (126), may affect the overall representation of cells in final samples and lead to skewed results in subsequent analysis of gene expression profiles.

Future research should further investigate the phenotype of pDCs to understand novel subsets, states, and their functions, and their potential association with the variability in inflammatory skin disease. It is also necessary to identify the breadth of cell types with IFN-I production capabilities, and IFN-I production pathways according to context- and patient-specific characteristics. Additionally, future research should clarify the mechanisms for pDC recruitment *in vivo* (38) and downstream effects after pDC activation (including subsequently activated cell types). The lifecycle and turnover of pDCs in lesional tissues also need to be determined, in addition to pDC fate after tissue migration. Biomarkers of pDC activity should also be identified to help investigate pDC function across entire biopsies, including the mechanisms for high IFN-I expression without pDC infiltration in the skin. Moreover, further research is needed on the mechanisms that lead to IFN-I education in lesional versus nonlesional skin (126). This is important to understand because local innate mediators of inflammation in the skin, such as myeloid cells, may help to explain why CLE can occur without ANAs and why it might be resistant to B-cell-targeted therapies (36). Lastly, the mechanisms by which pDCs suppress T-cell proliferation in peripheral blood, as observed *in vitro* in patients with SLE (36), should be investigated in target tissues where pDCs could function differently.

## 9 Conclusions

Studying the pathogenesis of SLE and CLE is inherently challenging owing to the complexity of the disease (5, 38). For example, in SLE there is a wide spectrum of clinical manifestations and diverse contributing factors, including disease activity, environmental triggers, and patient-specific characteristics such as genetic susceptibility (eg, high versus low polygenic risk, hormonal influences, and molecular endotypes) (2, 74, 135). Investigating IFN-I production by pDCs in the pathogenesis of SLE and CLE is also challenging. Several mechanisms of pDC activation exist that shape pDC responses (29), and pDCs may localize in target tissues in association with a strong IFN-I signature (126). However, IFN-I production involves the integration of multiple pathways and cellular processes in a way that is not yet fully deciphered (28, 29, 38).

Regarding pDCs, there is high phenotypical and functional heterogeneity of pDC subsets and states (29, 35). Activated pDCs have been reported to reduce their immunogenic functions in patients with SLE, including IFN-I production and antigen presentation (55, 125, 126). Fate switching has also been discovered whereby pDCs transition to a cDC2-like identity and function (97). Additionally, nonhematopoietic cells may also contribute to IFN-I production, thereby participating in the SLE disease process (125).

Our understanding of the role of pDCs, and their capacity for IFN-I production in the pathogenesis of SLE and CLE continues to grow and evolve. Continued research holds great promise for uncovering patient-specific responses to therapy, paving the way for the development of more targeted and effective treatments that can significantly enhance outcomes for individuals living with SLE and CLE (2, 13, 28).

## Glossary

ANA: antinuclear autoantibody
BDCA: blood dendritic cell antigen
BICLA: British Isles Lupus Assessment Group–based Composite Lupus Assessment
CCL: C-C-chemokine ligand
cGAS: cyclic guanosine monophosphate-adenosine monophosphate synthase
CLASI: Cutaneous Lupus Erythematosus Disease Area and Severity Index
CLE: cutaneous lupus erythematosus
CXCL: C-X-C motif chemokine ligand
DC: dendritic cell
DEJ: dermal-epidermal junction
DLE: discoid lupus erythematosus
FcγRIIa: Fc gamma receptor IIa
IC: immune complex
IFN: interferon
IFN-I: type I interferon
IFN-III: type III interferon
IL: interleukin
ILT7: immunoglobulin-like transcript 7
LLDAS: Lupus Low Disease Activity State
mAb: monoclonal antibody
MxA: myxovirus resistance protein A
NCF1: neutrophil cytosolic factor 1
NET: neutrophil extracellular trap
NF-κB: nuclear factor kappa B
PBMC: peripheral blood mononuclear cells
pDC: plasmacytoid dendritic cell
PDL: programmed death ligand
ROS: reactive oxygen species
SCLE: subacute cutaneous lupus erythematosus
scRNA-seq: single-cell RNA sequencing
Siglec: sialic acid-binding immunoglobulin-like lectin
SLE: systemic lupus erythematosus
STING: stimulator of interferon gene
TCF4: E protein transcription factor 4
TLR: Toll-like receptor
TNF: tumor necrosis factor
TYK: tyrosine kinase.
